# Supplementary material for: Prevalence of undiagnosed hypertension and risk assessment using a validated survey in community-based screening in Amman, Jordan
Source: PLoS One. 2026 Mar 23;21(3):e0345013. doi: 10.1371/journal.pone.0345013 (PMC13008244; doi:10.1371/journal.pone.0345013)
Supplement: S1 Table — (PDF) [file pone.0345013.s001.pdf]

S1

S1 Table

Table S1: 3-, 6-, 9-year Risk of Incident Hypertension by Baseline Factors and Total Risk Score.

Total points	Frequency (%)	3-year Risk, %	6-year Risk, %	9-year Risk, %
0	0	3	5	7
1	5(0.5%)	3	5	8
2	12(1%)	4	6	9
3	21(2%)	4	8	11
4	31(3%)	5	9	14
5	26(2%)	5	11	17
6	57(6%)	7	14	22
7	88(9%)	8	17	28
8	124(13%)	11	22	36
9	100(10%)	13	26	42
10	125(13%)	16	31	49
11	76(8%)	20	36	55
12	55(5%)	23	40	61
13	46(4%)	27	43	64
14	43(4%)	31	47	67
15	19(2%)	36	52	70
16	14(1%)	36	53	72
17	5(0.5%)	32	48	75
18	10(1%)	33	50	76
19	17(1%)	36	54	77
20	19(2%)	40	59	79
21	15(1%)	47	64	82
22	17(1%)	52	66	83
≥23	7(0.7%)	53	70	87
