# Supplementary material for: Prevalence of undiagnosed hypertension and risk assessment using a validated survey in community-based screening in Amman, Jordan
Source: PLoS One. 2026 Mar 23;21(3):e0345013. doi: 10.1371/journal.pone.0345013 (PMC13008244; doi:10.1371/journal.pone.0345013)
Supplement: S1 Questionnaire — (PDF) [file pone.0345013.s003.pdf]

جامعة البترا

Hypertension screening survey

Dear all：

This research aims to measure individuals′ knowledge of the factors that increase the risk of developing high blood pressure. In addition to conducting a quick check to ensure blood pressure level within normal limits. All information and data will be treated with strict confidentiality and data collected will only be used for medical analysis.

You can withdraw it at any time. Thank you for participating in this study.

This survey only takes up several minutes.

please answer all questions and measure blood pressure.

University of Petra-College of Pharmacy

- **I agree to participate** **Yes□ No□**
- Name (optional):_____________

Basic information:

1. Weight: _____ kg
2. Height: _____ cm
3. Telephone number in case if high blood pressure reading___________
4. Gender: □ Male □ Female
5. Date of Birth (DOB): _____ years
6. Current address:__________
7. Education:□ high schools□ diploma or less□ Bachelor □ Master □ PhD □
8. If you completed your degree education, was this education in one of the

following specializations? ; medical, (Medicine, pharmacy, nursing, dentistry, physical therapy, nutrition) □ Yes □ No

9. Do you eat fruits and vegetables on daily basis? At least one fruit, handful vegetable: □ Yes □ No

10. I like salty food (I eat more than a small spoon of salt) every day: □ Yes □ No

11. Income (monthly salary in Jordanian dinars):
Less than 250 dinars

□251-500 dinars

□501-750 dinars

□751-1000 dinars

□more than 1000 dinars

□I don't want to reveal my salary.

12. I have medical health insurance (government/private): □ Yes □ No

13. Marital status:

□Single

□Married

□Divorced

□Widowed

14. Are you a smoker?

□No, non-smoker.

□Yes, I am a current smoker (from what year did you start smoking)?____________

What type of smoking is it: (Circle the answer)?

regular cigarette
Or shisha.
Or electronic

Ex-smoker (number of years you have smoked)? □ Yes □ No___________

For how many years have you stopped smoking?________________
What type of smoking is it: (Circle the answer)?
regular cigarette?_______________
Or shisha.
Or electronic

15. The present or past history of the disease: □ Diabetes □ Hypertension □

Epilepsy □ cancer □ allergic asthma stroke □ heart disease□ gout□

Cholesterol □thyroid gland disease□ anemia□

□ other disease (please state) _____

16. Currently taking medication or supplements: _______________

17. Any of your parents and/or sibling suffer from hypertension? □ Yes □ No____

18. Your exercise frequency:

(Exercise Definition: duration 30 minutes) □ No □ once a month □ once a week

□ one week □ 3-5 times a day

19. Please write your resting blood pressure reading (no caffeine intake, including tea, green tea, coke, coffee, and no smoking, including shisha, cigarette, vape, all these she be stopped at least 30 minutes before blood pressure measurement): _______________

systolic blood pressure -the upper reading (SBP)

____

diastolic blood pressure – the lower reading (DBP)

Thank you for your participation
I wish you good health
