# Supplementary material for: Prevalence of undiagnosed hypertension and risk assessment using a validated survey in community-based screening in Amman, Jordan
Source: PLoS One. 2026 Mar 23;21(3):e0345013. doi: 10.1371/journal.pone.0345013 (PMC13008244; doi:10.1371/journal.pone.0345013)
Supplement: Attachment — Submitted filename: Response to reviewers.docx [file pone.0345013.s004.docx]

**Point-by-Point Response to the Reviewers**

**Response to Reviewers**

Manuscript ID: PONE-D-25-06643

Title: Use of a Risk Assessment Questionnaire to Identify Individuals with Hypertension in a Community-based Screening in Amman, Jordan

We thank the Editor and Reviewers for their time and constructive comments, which have helped us to improve the quality and clarity of our manuscript. Below we provide a detailed, point-by-point response. Reviewer comments are reproduced in bold, followed by our responses in regular font. All changes have been incorporated in the revised manuscript.

**Response to Editor**

- E1: Please ensure that your manuscript meets PLOS ONE's style requirements, including those for file naming. The PLOS ONE style templates can be found at <https://journals.plos.org/plosone/s/file?id=wjVg/PLOSOne_formatting_sample_main_body.pdf> and <https://journals.plos.org/plosone/s/file?id=ba62/PLOSOne_formatting_sample_title_authors_affiliations.pdf>

Response E1: All manuscript revised, style now according to journal requirement.

- E1: Please include a complete copy of PLOS’ questionnaire on inclusivity in global research in your revised manuscript. Our policy for research in this area aims to improve transparency in the reporting of research performed outside of researchers’ own country or community. The policy applies to researchers who have travelled to a different country to conduct research, research with Indigenous populations or their lands, and research on cultural artefacts. The questionnaire can also be requested at the journal’s discretion for any other submissions, even if these conditions are not met. Please find more information on the policy and a link to download a blank copy of the questionnaire here: https://journals.plos.org/plosone/s/best-practices-in-research-reporting. Please upload a completed version of your questionnaire as Supporting Information when you resubmit your manuscript.

Not relevant

- E1: We note that the grant information you provided in the ‘Funding Information’ and ‘Financial Disclosure’ sections do not match. When you resubmit, please ensure that you provide the correct grant numbers for the awards you received for your study in the ‘Funding Information’ section.

We are arranging that in resubmission, we will confirm the funding information.

- E1: Thank you for stating the following financial disclosure: “H.B. was partly supported by the National Institutes of Health through grants UL1 TR001860. The content is solely the responsibility of the authors and does not represent the official views of the National Institutes of Health” Please state what role the funders took in the study. If the funders had no role, please state: "The funders had no role in study design, data collection and analysis, decision to publish, or preparation of the manuscript." If this statement is not correct you must amend it as needed. Please include this amended Role of Funder statement in your cover letter; we will change the online submission form on your behalf.

All correct statements of funders and their roles are mentioned, in cover letter and manuscript and submission system.

- E1:Thank you for stating in your Funding Statement: “H.B. was partly supported by the National Institutes of Health through grants UL1 TR001860. The content is solely the responsibility of the authors and does not represent the official views of the National Institutes of Health” Please provide an amended statement that declares *all* the funding or sources of support (whether external or internal to your organization) received during this study, as detailed online in our guide for authors at http://journals.plos.org/plosone/s/submit-now. Please also include the statement “There was no additional external funding received for this study.” in your updated Funding Statement.

Included

- E1:Please include your amended Funding Statement within your cover letter. We will change the online submission form on your behalf.

Included

- E1:In the online submission form, you indicated that “The datasets used and analyzed for this study are available from the first author upon reasonable request.” All PLOS journals now require all data underlying the findings described in their manuscript to be freely available to other researchers, either 1. In a public repository, 2. Within the manuscript itself, or 3. Uploaded as supplementary information. This policy applies to all data except where public deposition would breach compliance with the protocol approved by your research ethics board. If your data cannot be made publicly available for ethical or legal reasons (e.g., public availability would compromise patient privacy), please explain your reasons on resubmission and your exemption request will be escalated for approval.

We chose: 3. Uploaded as supplementary information.

- E1:Please include captions for your Supporting Information files at the end of your manuscript, and update any in-text citations to match accordingly. Please see our Supporting Information guidelines for more information: <http://journals.plos.org/plosone/s/supporting-information>.

The manuscript has been entirely revised to meet the formatting requirements specified by the journal.

**Response to Reviewers**

**Reviewer #1:**

Overall, good paper but major improvements can be made to improve overall output

However, there are a few clarifications:

- R1: the objective states "identify undiagnosed cases of hypertension in the general public using a risk-scoring tool for elevated BP and hypertension" - it wasn't clear in the methods how the use of risk scoring tool identified undiagnosed cases of hypertension - the methods essentially said that the study got the risk score and got the BP (and not used the risk score to identify undiagnosed hypertension as the objective and title imply that being said may be the title and objective should reflect that was an identification of the prevalence of hypertension and estimating risk using the risk-scoring tool.

We appreciate this important clarification. You are correct that our original phrasing was misleading. We have revised both the title and objective to accurately reflect our study design:

Revised Title: "Prevalence of Undiagnosed Hypertension and Risk Assessment Using a Validated Questionnaire in Community-based Screening in Amman, Jordan"

Revised Objective: "The current study aimed to identify the prevalence of undiagnosed hypertension and assess future hypertension risk using a validated risk-scoring tool in the general public in Amman, Jordan."

We have also clarified in the abstract and methods that we conducted BP measurements to identify current undiagnosed hypertension AND separately used the risk-scoring tool to assess future hypertension risk among participants. This dual approach provides both immediate clinical value (identifying those who need treatment now) and preventive value (identifying those at high risk who need monitoring and lifestyle interventions).

Standardization of BP measurement device

- R1: There was no standardization of the BP app used - please indicate the model and why this was used - cite references that it is validated for office use.

Thank you for highlighting this important methodological detail. We have added the following information to the Methods section:

Pharmacy students helped to measure and record resting BP using the Omron™ BP measuring electronic machine (Model: LM Model, Approval No: IND/09/21/814, Omron Healthcare, Osaka, Japan). This model has been validated for clinical use according to international protocols and is recommended by the European Society of Hypertension for office BP measurements. Reference is ESH (European Society of Hypertension), also OMRON products are validated by the American Medical Association, see <https://omronhealthcare.com/clinical-validation>

- R1: There was no standardization on the times BP was taken. Please indicate reason why this was not protocolized.

We acknowledge this limitation and have expanded our discussion (page-14) and amended protocols in the Methods section (page-5):

"BP was generally measured once; if the result was abnormal (SBP ≥130 mmHg or DBP ≥80 mmHg), a second reading was taken after a 1-2 minute rest period, and then the two readings were averaged. Infrequently, three readings were taken and averaged when there was >10 mmHg difference between the first two readings. While standardized multiple readings at fixed intervals (e.g., 3 readings at 1-minute intervals) are ideal for BP diagnosis, this approach was not feasible (extra burden and inconvenient) in our community-based screening setting due to time constraints and the large number of participants. This pragmatic approach balances accuracy with feasibility in resource-limited community settings, though we acknowledge this as a limitation."

We have also added this to the Limitations section: "The lack of a standardized protocol for multiple BP measurements at fixed intervals may have introduced measurement variability, though we attempted to minimize this by taking additional readings for abnormal results. "

Results separated systolic and diastolic hypertension.

- R1: Is there a reason for this? Why not use the standard definitions only for hypertension as the summary result?

For the sake of Data Granularity, in our cohort, some participants met criteria for only SBP or DBP elevation. Separating these allowed us to explore whether the risk score predicted one subtype more strongly. We agree that the standard definition of hypertension (SBP ≥140 or DBP ≥90) is critical for clinical diagnosis, and we aimed to provide additional nuance for research purposes. For transparency, we included the hypertension outcomes (SBP ≥140 or DBP ≥90).

Analytical statistics for high risk score vs. current hypertension

- R1: Suggest to do analytical statistics correlating HIGH risk score (>17) with incident hypertension at present (BP >140/90). This is a simple 2x2 table.

We thank the reviewer for this excellent suggestion. We have added the following analysis to the Results section:

To examine the relationship between high-risk scores and current hypertension status, we conducted a 2x2 contingency table analysis.

Participants with risk scores ≥17 (n=39) were those who had current hypertension (BP ≥140/90 mmHg), the high-risk and lower-risk participants are summarized in Table 5.

analytics of the profile of those with HIGH score >17

- R1: Suggest to include analytics of the profile of those with HIGH score >17, and use that as jumping point for meaningful discussion on the timing of screening using the questionnaire.

Those were categorized as high risk at the time of screening. We have added a new subsection in the Results:

Characteristics of High-Risk Participants (Score ≥17)

Among the 39 participants with risk scores ≥17, the following characteristics were observed:

- Mean age: 58 years (SD ±9)
- Female: 38%
- Current smokers: 29%
- Family history of hypertension: 72%
- BMI ≥30: 54%
- Diabetes: 31%
- No regular exercise: 67%

Improve discussion based on analytical analysis

- R1: Improve the discussion to include suggested analytical analysis.

We have substantially revised the Discussion section.

Our analysis revealed that participants with risk scores ≥17 were added extensively.

Based on our findings, we propose a risk-stratified screening approach:

High risk (score ≥17):

Moderate risk (score 11-16):

Low risk (score <11):

This approach could optimize resource allocation in resource-limited settings while ensuring timely detection of hypertension among those at highest risk."

Additional limitations

- R1: Suggest to include recall bias in the limitations of the study (there were questions about salt intake and exercise) and bias of the methods of BP measurement.

We agree with you and have expanded the Limitations section:

"Additional limitations include potential recall bias in self-reported variables such as exercise habits, which may lead to underestimation of these risk factors. Furthermore, the variability in BP measurement methods, including the lack of standardized timing between readings and potential differences in cuff positioning or participant preparation across multiple student measurers, may have introduced measurement bias. Although students received training, inter-observer variability cannot be excluded."

Grammatical correction

- R1: In conclusion grammatical error: switch "unlikely aware" to "likely unaware"

Thank you for catching this issue. We have corrected the sentence to read: "Almost 70% of study participants met the AHA criteria for a diagnosis of prehypertension or hypertension but they were likely unaware of their (pre)hypertension status."

Abstract revision

- R1: Improve abstract based on ALL comments above.

We have comprehensively revised the abstract to address all points. See the revised Abstract.

We believe these revisions substantially strengthen the manuscript and address all of the reviewers' concerns. We appreciate the opportunity to improve our work based on this constructive feedback. We will be glad to listen to additional advice if any.

**Reviewer #2:** Here are a few comments for the attention of the Authors:

After reading this part of the methods “Students participated in this study and also distributed questionnaires randomly to their family members and friends/neighbors and were instructed to invite persons without known hypertension” I am still confused as to what kind of recruitment strategy was used to select the students (I see serious selection bias)

- R2: Its is not very clear how the study process went. Questionnaires were administered first? Then they came to where to get BP measured? Please clarify these in the write up

We acknowledge this important concern. We used convenience sampling through pharmacy students at the University of Petra. Students were recruited voluntarily from the Faculty of Pharmacy and then distributed questionnaires to their family members, friends/and neighbors. We recognize this can introduce selection bias, which we now explicitly acknowledge in the Limitations section (page 11): "Lastly, a convenience sample may not represent a target population, such as the wider university family, Amman, or Jordanian population."

- R2: "It's not very clear how the study process went. Questionnaires were administered first? Then they came to where to get BP measured?"

We have clarified the process in the Methods section.

The questionnaire administration and BP measurement co-occurred during the same visit. Pharmacy students helped participants’ complete questionnaires and measured their BP at the same time.

We added this clarification page 5: "Data collection occurred in a single visit where participants completed the questionnaire with assistance from trained pharmacy students who also measured their blood pressure during the same session."

- R2: "The protocol followed in assessing the blood pressure doesn't seem quite standard. Why are some people measured once, some twice and some thrice? Meanwhile, what is the meaning of an elevated BP reading?"

We acknowledge this limitation. The protocol was:

- First measurement taken for all participants
- If the reading was elevated (SBP ≥120 or DBP ≥80 mmHg), a second reading was taken
- Rarely, if readings were higher than normal, a third measurement was taken
- "elevated or high" refers to any reading outside the normal range (SBP <120 and DBP <80)

We recognize this is not the medical usual standard (multiple readings on different days) but was a compromise for a large community-based screening. This is acknowledged in our Limitations section (lines 302-305).

- R2: "The sentence about score ≥17 points... shouldn't be part of statistical analyses"

We agree. This information about the risk scoring system should be moved to the Risk Assessment Tool section where the scoring system is described. The minimum obtainable score is 0 points, and the maximum is approximately 30 points, which we clarified in that section. These sentences have been removed from statistical analysis.

- R2: "No need repeating the entire results in the Table and the narration". Results of those in Amman and other provinces is not relevant.

We agree that some repetition exists. We revised the text to highlight key findings, referring readers to tables for complete details. We have revised based on the reviewer’s comments. Focused on Amman results only.

- R2: "Why is there a citation at the last sentence of the results section?"

The citation (reference 5) appears to be misplaced. Results sections should not contain citations. This reference was deleted.

*R2: "Conclusion should be kept short. Most of the content there should go to discussion."*

We agree. The current Conclusion section contains discussion elements. We propose condensing it to focus only on:

Key finding: ~70% met criteria for prehypertension/hypertension

Main implication: Need for community-based screening programs

Conclusion was shortened per reviewer suggestion.
