# Supplementary material for: Prevalence of undiagnosed hypertension and risk assessment using a validated survey in community-based screening in Amman, Jordan
Source: PLoS One. 2026 Mar 23;21(3):e0345013. doi: 10.1371/journal.pone.0345013 (PMC13008244; doi:10.1371/journal.pone.0345013)
Supplement: Attachment — Submitted filename: Response_to_reviewers_auresp_3.docx [file pone.0345013.s006.docx]

**Point-by-Point Response to the Reviewers**

Thank you for the revision—overall, the paper is now publishable. Only minor points remain:

Abstract clarity: The “Methods” sentence is still grammatically unclear and should be rewritten to

describe exactly what was done (BP measurement + risk score survey in the same visit), without

redundancy.

Study process and recruitment: Keep the description simple and explicit (how students were recruited;

how participants were approached; questionnaire and BP measured in the same session). Maintain

cautious language about generalizability due to convenience sampling.

BP measurement protocol: Ensure consistent definitions and thresholds across the manuscript

(normal/elevated/stage categories), and keep the pragmatic repeat-reading approach clearly

described. Emphasize the limitation of non-standardized repeated readings and potential interobserver variability.

Student training/proficiency: Since proficiency testing was not verified, state this clearly as a limitation

(training provided, but no formal competency assessment).

Risk tool performance reporting: Make sure the contingency analysis outputs (e.g., OR and screening

performance metrics) are presented consistently in the Results/Tables and interpreted conservatively

(screening/stratification utility rather than diagnostic certainty)

• Abstract Methods: Clear sequential description of what was done

• Recruitment: Explicit student volunteer process stated

• BP protocol: Pragmatic repeat-reading approach with acknowledged limitations • Training: Clearly stated as limitation (no proficiency testing)

• Risk tool: Conservative language emphasizing screening/stratification utility

Figures were made available in 300dpi and tiff

**Response to Reviewers**

Manuscript ID: PONE-D-25-06643

Title: Use of a Risk Assessment Questionnaire to Identify Individuals with Hypertension in a Community-based Screening in Amman, Jordan

We thank the Editor and Reviewers for their time and constructive comments, which have helped us to improve the quality and clarity of our manuscript. Below we provide a detailed, point-by-point response. Reviewer comments are reproduced in bold, followed by our responses in regular font. All changes have been incorporated in the revised manuscript.

Review Comments to the Author Please use the space provided to explain your answers to the questions above. You may also include additional comments for the author, including concerns about dual publication, research ethics, or publication ethics. (Please upload your review as an attachment if it exceeds 20,000 characters) Reviewer #1: All previous comments were addressed but there are more comments which I would like to highlight

**Response to Reviewers**

**Reviewer #1:**

All previous comments were addressed but there are more comments which I would like to highlight

- R1: Label Abstracts components accordingly for easier reading. Background and Objectives. Methods. Results. Conclusion

1. Abstract structure: We have restructured the abstract with clear labels:

•	Background and Objectives

•	Methods

•	Results

•	Conclusion

- R1: 2. Abstract can still be improved to be more clear

The abstract has been revised for clarity, focusing on key findings and removing redundancy.

- R1: 3. Include a flowchart of the participants (from questionnaire distribution until final analysis)

Participant flowchart: A CONSORT-style flowchart has been added as Figure 1, showing questionnaire distribution (n=1000) → responses received (n=932) → excluded for incomplete data (n=68) → final analysis (n=932).

Added flowchart figure per reviewer request

- R1: 4. Be EXACT in the questionnaires distributed, do NOT say "approximately 1000..."

Exact questionnaire numbers: Corrected to: "A total 1000 questionnaires were distributed" (removed "approximately").

- R1: 5. Table 3 is a bit too distracting. Suggest to append and write in text form the important findings from this table

Table 3: Table 3 has been moved to supplementary materials. Key findings are now presented in text and Figure 2

- R1: 6. Suggest to append Fig 1 as well

Figure 1: The original Figure 1 (risk distribution) has been added

.

- R1 7. What is the basis for "risk stratified screening approach". Also this statement is wrongly placed as it should be a point for discussion rather than a result.

This section has been moved from Results to Discussion

- R1 8.Re Contingency Table, pls add important parameters like OR and Sn/Sp of the risk strat tool for current hypertension

Added to Table 4:

OR: 19.3 (95% CI: 9.2-40.4, p<0.001), Sensitivity: 62.8%, Specificity: 96.2%, PPV: 64.8%, NPV: 95.8%

- R1: 9. Briefly discuss the current Jordan policy on hypertension screening if there is. The discussion sounds like there is currently no program in place.

Added to Discussion: "Jordan's National Strategy for Non-Communicable Diseases recommends opportunistic BP screening in primary care settings. However, systematic community-based screening programs remain limited, highlighting the need for our approach."

- R1: 10. Include the MODEL of the OMRON BP App

Added: "Omron HEM-907XL IntelliSense Professional"

- R1: 11. Was there an assessment of proficiency test done on the students or teaching was assumed to be effective without proper post teaching assesment?

No

Student proficiency was not verified

- R1: 12. Discussion can still be improved to highlight the importance of the risk factors. Focus the discussion on the identified risk factors in the study and the prevalence of these as well and how it can be addressed.

Enhanced Discussion focuses on modifiable risk factors: "The high rates of modifiable risk factors, namely physical inactivity (62%), smoking (38%), and obesity (21%), show that there are high opportunities for targeted interventions. The existing tobacco control programs in Jordan will be of special benefit to this group."

- R1: 13. Suggest to discuss about the "high risk scorers" without current hypertension

Added to Discussion: "Notably, 16/39 (41%) high-risk scorers did not have current diagnosed hypertension, representing a significant prevention opportunity. These individuals require intensive lifestyle counseling and frequent BP monitoring."

- R1: 14Suggest to remove sentence on health information overload in the conclusion as this was never discussed previously in the paper

This phrase has been removed from the Conclusion.

- R1:15. Focus on the basics of research. The objective should answer your clinical/epidemiologic question, the methods should justify the answering of the objective -> the conclusion should answer the objective

•	Objective: "This study assessed the prevalence of undiagnosed hypertension through blood pressure measurement and evaluated future hypertension risk using a validated survey in a community setting."

•	Methods: Aligned to address both prevalence and validation

•	Conclusion: "Our community screening revealed a high prevalence of undiagnosed hypertension (20.6%) and identified a substantial proportion at high risk for future hypertension"

All changes are highlighted in the revised manuscript.
